# Factors influencing healthcare-seeking behavior and its process analysis among ethnic minority residents: a qualitative study in rural China

**DOI:** 10.3389/fpubh.2025.1627392

**Published:** 2025-06-20

**Authors:** Shu Wei, Haifeng Long, Yu Tian

**Affiliations:** ^1^College of Humanities and Social Development, Northwest Agriculture and Forestry University, Yangling, China; ^2^College of Humanities and Social Development, Northwest Agriculture and Forestry University, Yangling, China; ^3^School of Public Affairs, Zhejiang Shuren University, Hangzhou, China

**Keywords:** healthcare-seeking behavior, social capital, physician-patient trust, ethnic medicine, social norms

## Abstract

**Background:**

Healthcare-seeking behavior is a crucial foundation for improving the rational use of healthcare resources and enhancing community health. Existing studies have predominantly focused on quantitative analyses of healthcare-seeking choices and their determinants based on the Homo Economicus assumption, while neglecting the analysis of sociocultural processes underlying healthcare-seeking behavior.

**Methods:**

This study employed participant observation and in-depth interviews to investigate thirty-two residents from ethnic minority rural areas, with selected typical cases subjected to focused discussion. Based on the theoretical perspective of social capital, this paper explores and analyzes the healthcare-seeking behavior and its underlying logic in ethnic minority rural areas.

**Results:**

The findings suggest that institutional trust and interpersonal trust influence patients’ choices between formal and informal medical systems. Medical care information, as a prerequisite for patients’ decisions, flows differently within ethnic relationship networks and community social networks. Institutional and cultural norms collectively influence healthcare service behaviors and provide support for patients’ healthcare-seeking behavior.

**Conclusion:**

The adjustment of social medical policy should be guided by the advantages of the formal medical system and the informal medical system, so that the primary medical system can provide high-quality medical services for local residents.

## 1 Introduction

According to data from the China Health Statistics Yearbook (1), both the proportion of outpatient visits and hospital admissions in primary healthcare institutions have demonstrated persistent contraction in recent years, indicating emerging behavioral shifts in healthcare-seeking decisions among rural residents under contemporary socioeconomic conditions.

Healthcare-seeking behavior refers to the series of actions individuals undertake when perceiving physical discomfort or abnormal conditions, including the selection of medical institutions, healthcare providers, and medications, to alleviate suffering or cure diseases. Research on healthcare-seeking behavior primarily focuses on four dimensions:

First, population-specific investigations: For adolescent populations, critical influencing factors include medical costs and demographic characteristics (2); economic status, health insurance, and healthcare accessibility significantly impact rural older adults populations (3); Second, regional explorations: Studies on factors influencing hospital selection among residents in Indonesia (4) and Uganda (5), along with analyses of influencing factors and measurement approaches regarding healthcare facility selection among urban youth in Chinese communities (6); Third, investigations into influencing factors of healthcare choices: The role of individual enabling resources in healthcare decision-making (7); the impact of medical resource accessibility on patient choices (8); and the influence of online medical consultations on healthcare-seeking behavior (9); Fourth, sociocultural implications: Evaluations of healthcare policy effectiveness through patient choice patterns (10).

Existing research on healthcare-seeking behavior has primarily developed along two analytical pathways: The first examines influencing factors through the rational choice theory framework, including evaluations of medical system safeguard effectiveness (11), healthcare service quality (12), and treatment-related costs (13). The second investigates cultural determinants through sociocultural theoretical lenses, analyzing constructs such as gender bias in clinical decision-making (14), ethnomedical paradigms (15), and illness-related shame (16).

A critical examination of current medical practices in China reveals that while the aforementioned research content and theoretical frameworks have provided insightful analyses, notable deficiencies persist. Primarily regarding research scope, existing studies have insufficiently addressed the ethnic characteristics inherent in China’s traditional medicine and ethnomedicine systems within rural social contexts of ethnic minorities. Secondly, in terms of analytical approaches, predominant scholarship has predominantly employed the homo economicus assumption to interpret healthcare-seeking behaviors, yet crucially neglects to incorporate agent-centric perspectives in explicating the social determinants and constitutive processes underlying such health-seeking decision-making within societal environments.

The theory of social capital was initially formulated by Bourdieu as “the aggregate of actual or potential resources embedded within institutionalized networks of mutual recognition” that facilitates social actions among community members or collectives (17). This seminal work accentuates social capital’s inherent characteristic as a class-based resource and its consequential impact on individual social agency. From a functionalist perspective, Coleman conceptualized social capital as social structural features—including trust, norms, and obligations—that enable individual or collective action (18). Putnam’s analytical framework further identified trust, norms, and social networks as the core constituents of social capital (19). Lin’s investigation into social capital mechanisms revealed that its acquisition fundamentally relies on direct or mediated social relationships (20). Empirical studies in the Chinese context have substantiated social capital’s critical influence on actors’ behavioral processes and situational contexts (21, 22). Sociopractically, China’s traditional ties-based social fabric endows social capital with significant operational potency in socioeconomic engagements (23).

Social capital, as a crucial element within social contexts, influences health-related behaviors through specific causal mechanisms in societal structures (24), demonstrating significant correlations with health behaviors. This multidimensional construct exerts both direct effects on population health outcomes and indirect impacts through mediating mechanisms such as individual income stratification (25). Empirical studies identify positive associations between social capital and health indicators, with particular emphasis on the more pronounced health effects of collective social capital (26, 27). Notably, interpersonal trust - a core component of social capital - demonstrates significant positive influences on self-rated health status and healthcare-seeking behaviors (28, 29). Although extant studies have empirically demonstrated the correlational relationship between social capital and healthcare-seeking behavior through quantitative analyses, there remains a paucity of scholarly discussion regarding the micro-level mechanisms through which social capital operationalizes its influence on healthcare-seeking behavior patterns.

Building upon this theoretical framework, this study addresses the following research question: How do healthcare-seeking choices among residents in ethnic minority villages in China emerge within their social context, and what social factors influence these behaviors? By integrating social capital theory, this paper investigates the behavioral processes and underlying logic guiding healthcare-seeking decisions among rural ethnic minority populations.

## 2 Methodology

### 2.1 Research design

This study employed a qualitative research methodology. A non-probability sampling method ensured diversity and representativeness among participants regarding healthcare institution selection, cultural backgrounds, and disease typology. Specific data collection methods included participant observation in medical institutions, in-depth interviews with research participants, and online follow-up surveys. Field investigations spanned from December 2022 to October 2023, with intensive fieldwork concentrated over approximately two months. The qualitative approach was selected for its capacity to thoroughly explore participants’ healthcare trajectories and social determinants influencing medical processes (30). Depth interviews were chosen for their efficacy in elucidating nuanced descriptions of healthcare-seeking behaviors (31), with average durations exceeding three hours per session for both interviews and longitudinal surveys.

The field investigation was conducted in Huangguoshu Town, Guizhou Province, China. Survey sites included the Community Health Center, village clinics, and county-level medical institutions governing the township. Huangguoshu Town represents a typical ethnic minority settlement area, inhabited by Buyi, Sui, Gelao, Miao, Dong, and Hui ethnic groups, which have formed natural ethnic villages. The town’s healthcare system comprises two categories: (1) the formal medical system, consisting of 2 community health centers and 10 village clinics; and (2) the informal medical system, which includes local practitioners of folk medicine and ethnic medical practices.

### 2.2 Participants

Thirty-two healthcare-seeking individuals and medical professionals participated in the final study, encompassing diverse ethnic identities, occupational backgrounds, disease types, and socioeconomic statuses. Participants ranged in age from 29 to 69 years, with the majority clustered between 30 and 40 years. Due to textual space constraints, eight representative cases were selected for detailed analysis. Table 1 presents comprehensive healthcare-seeking behavioral data from these core cases. Although the core case sample size was relatively small (N = 8), recurring thematic patterns emerged consistently in final interviews, though larger samples might reveal additional societal influencing factors and variations in care-seeking trajectories. This data sufficiency aligns with the study’s exploratory objective of understanding sociostructural influences on healthcare-seeking populations and their decision-making processes. In-depth analysis of core cases revealed that participants across ethnic groups universally demonstrated trust in traditional medical practices when selecting healthcare modalities.

**Table 1:** Healthcare-seeking behaviors of core cases (*N* = 8).

ID	Gender	Age	Social identity	Disease type	Healthcare choice
1	Female	61	Farmer	Hypertension	Village clinic and community health center
2	Male	33	Merchant	Gastroenteritis	Community health center
3	Female	54	Folk healer	—	—
4	Female	30	Tourist	Common cold	Community health center
5	Male	33	Company employee	Fever	Community health center
6	Male	33	Non-local resident	Post-injury recovery	Ethnic medicine
7	Male	51	Buyi ethnic healer	—	—
8	Female	53	Hospital nurse	—	—

### 2.3 Ethics statement

The study followed the principles of respect for the individual, benevolence and impartiality as set out by the Institutional Review Board (32). The process by which researchers establish contact and conduct interviews with study participants primarily follows two distinct pathways. The first pathway involves collaboration with local government entities and staff from formal healthcare institutions to access respondent populations. The primary interview methodologies employed encompass: (1) Focus group interviews: Structured discussions convened with physicians and patient cohorts to examine healthcare-seeking behaviors; and (2) Longitudinal Investigation: Assigning a dedicated researcher to establish a robust rapport with pivotal case studies and conduct systematic follow-up assessments. The alternative pathway entails researcher-initiated engagement through observation methodology, involving autonomous conduct of interviews and real-time documentation with healthcare service recipients during observational field studies.

Additional safeguards were implemented during data collection, along with establishing procedures to handle medical safety and ethical concerns. Research data underwent translation and transcription, with researchers offering summarized paraphrases throughout data collection and analysis rounds to protect the privacy of the research subjects. Given the potential impact of the in-depth interviews method on participants’ responses, the discomfort of some patients with records of the document, and the necessity to protect participants’ privacy, this study employed an oral consent procedure to obtain informed consent from all participants prior to conducting interviews and case studies. This procedure aligns with the Declaration of Helsinki and has been approved by the Northwest A&F University Research and Ethics Committee (No. S202310712460).

## 3 Results

### 3.1 Theme1: healthcare-seeking behavior under the dominance of dual trust orientation

This theme study investigates the specific trust attitudes influencing patients’ healthcare-seeking behaviors. The pluralistic medical system in ethnic community provides patients with a spectrum of healthcare options, encompassing both formal and informal medical institutions. Patients’ choices among these medical systems are shaped by their distinct trust dispositions, which typically manifest as a superimposition of institutional trust and interpersonal trust. However, divergent trust orientations assume dominant roles in guiding selections across different medical systems.

Patients’ preference for formal medical institutions predominantly stems from institutional trust in the rural medical and health system. The formation of this trust attitude relies on both the implementation processes of formal medical institutions and their efficacy in safeguarding patients’ health outcomes.

*“As an older adult individual with chronic hypertension requiring medication, I undergo periodic health examinations and prescription renewals arranged by the village clinic physician. Today, I consulted a doctor in the village clinic regarding chest discomfort, and he recommended further diagnostic procedures at the central hospital. The village clinic suffices for minor ailments due to its proximity and our established familiarity with the physician. He frequently accommodates our medical needs, for which I maintain profound gratitude. The central hospital also coordinates referrals to county-level hospitals, where geriatric specialists provide hypertension management guidance and medication protocols. We benefit substantially from institutional healthcare support, including complimentary diagnostic services with nominal medication fees.” (Participant 1).*

Institutional trust refers to the process wherein an audience develops trust in a specific institution and acts in accordance with institutional regulations under the guidance of this trusting attitude. This constitutes a transition from macro to micro levels and from psychological attitudes to behavioral practices. The institutional foundation of trust encompasses both regulatory frameworks and the organizations and roles responsible for implementing these rules (33). Within rural societal contexts, formal medical institutions include not only fundamental healthcare systems such as medical resource allocation mechanisms and health insurance/security systems but also implementation entities like community health centers, village health clinics, and their personnel.

Participant 1’s hypertension diagnosis was completed through a free physical examination at Community Health Center, coordinated by village healthcare practitioners. Concurrently, the treatment process was conducted under the guidance of both the Community Health Center and the village health clinic. The entire diagnosis-treatment procedure, executed in compliance with healthcare policy regulations and facilitated by formal medical institutions and their staff, established the foundation for Participant 1’s institutional trust in formal medical regulations and interpersonal trust in village medical practitioners.

As a recipient of the ethnic community’s formal medical system, Participant 1 received institutional support and assistance. Through synthesizing their disease management experience, they developed both institutional trust toward the ethnic community’s formal medical system and interpersonal trust toward healthcare workers. The formative process of this trust attitude reveals that grassroots medical institutions constituted critical determinants influencing Participant 1’s healthcare trajectory. Consequently, institutional trust played a dominant role in Participant 1’s selection process regarding formal medical institutions.

Comparing with participant1, participant 2’s clinical experience at the Community Health Center proved unsuccessful, which constituted the primary catalyst for his development of distrust toward the institution.

*“The day after consuming alcohol, I experienced stomach pain and diarrhea. During a visit to the Community Health Center, the physician perfunctorily inquired about my symptoms, recent activities, and diarrheal frequency before dismissing the condition as minor. I was prescribed medication and discharged. However, the symptoms persisted despite adherence to the treatment. That evening, the abdominal pain became unbearable, necessitating emergency transportation by a family member to the county hospital (a tertiary medical facility), where I was diagnosed with ulcerative colitis. I voiced strong dissatisfaction with the initial consultation, which involved a perfunctory examination lasting only a few minutes prior to inappropriate discharge. Medical practice demands rigorous diligence—such negligence constitutes endangerment of life for financial expediency. Consequently, I have discontinued utilization of Central Hospital for healthcare services.” (Participant 2).*

In contrast to Participant 1’s institutional trust in the formal medical system of ethnic community, Participant 2 developed skepticism toward the system’s efficacy during his engagement with its institutional framework. His illness and disease remained unalleviated through interactions with the community’s formal medical infrastructure, fundamentally undermining his confidence in the system’s healthcare delivery outcomes. This sentiment aligns with Participant 7 (a medical practitioner at Community Health Center) stating during interviews: *“Patients typically discontinue utilizing hospital services when encountering unclear diagnoses, issues with medical insurance reimbursement, and errors in the treatment process. While we physicians strive for universal patient satisfaction, operational realities impose practical constraints.”* Through rational evaluation of the formal medical system’s cost–benefit ratio and implementation, Participant 2, as a healthcare consumer within this institutional framework—formulated concrete distrust, subsequently electing to disengage from Community Health Center for medical care. When patients interface with formal medical institutions, their experiential evaluation of diagnostic processes and health insurance mechanisms crystallizes into specific trust orientations toward institutionalized healthcare systems. These trust dispositions subsequently govern healthcare-seeking behaviors.

Unlike formal medical systems characterized by external regulatory oversight and professional authority, informal medical institutions operate without such structural safeguards. In these contexts, patients base their healthcare decisions on interpersonal trust cultivated through direct interactions with institutional members.

Participant 3 operates a non-governmental healthcare provider within the township, employing treatment methodologies derived from self-directed study of experiential knowledge acquired through prolonged personal illness. Notably, Participant 3 lacks formal physician’s licensure. The services provided encompass basic medical consultations and limited pharmaceutical distribution, primarily targeting older adults villagers experiencing transportation barriers to hospital access.

*In my capacity as a community health volunteer, I primarily address minor ailments and dispense basic medications to older adult villagers, particularly those facing mobility limitations and challenges in accessing hospital services. When the designated physician at the village clinic is unavailable, patients frequently seek medical assistance from me. My extended hospitalization during treatment for liver cirrhosis provided substantial clinical exposure, though I maintain strict referral protocols, directing patients with severe conditions to formal medical facilities. My distinctive contribution lies in psychosocial support through therapeutic communication. Having personally endured prolonged illness, I possess acute empathy for the sick role experience. Recognizing the time constraints of institutional healthcare providers in patient-provider interaction, I consciously alleviate patient distress through reassurance and health counseling, emphasizing illness normalization and recovery prognosis. (Participant 3).*

Interpersonal trust refers to the cognitive judgment made by the trustor regarding the competence and reliability of the trustee (34). Participant 3 derives patients’ interpersonal trust through two distinct mechanisms. Primarily, her capacity for active listening and empathetic understanding facilitates patients’ formation of reliability assessments. Disease is not all physiological, but also includes the connection between the occurrence of disease and the external environment, namely illness (35). While conventional therapeutic paradigms prioritize physiological rehabilitation, the restorative potential of narrative reconstruction emerges as crucial in mitigating life-world disintegration caused by illness. Participant 3’s therapeutic engagement satisfies patients’ need for social suffering narration, thereby fostering identity validation that reinforces perceptions of clinical reliability.

Secondarily, trust in Participant 3’s medical expertise stems from her accumulated localized knowledge through illness management practices. This knowledge system, comprising medical habitus, community support networks, and emotional reciprocity mechanisms (36), provides the technical foundation for delivering enhanced clinical experiences. Patients’ subsequent healthcare-seeking behaviors manifest as emotional choices predicated on this established interpersonal trust.

The analytical framework reveals both convergence and divergence in trust logics across medical systems. A dual trust mechanism - integrating institutional and interpersonal dimensions - critically informs healthcare decision-making. In formal medical institutions governed by bureaucratic rationality (e.g., Community health centers and village clinics), institutional trust predominates through standardized regulatory assurances. Conversely, informal medical systems operating under socio-cultural logics prioritize interpersonal trust in individual practitioners when directing healthcare choices. These complementary trust paradigms collectively constitute the structural determinants of medical pluralism in contemporary healthcare-seeking behaviors.

### 3.2 Theme2: the selection of healthcare services within the relational network field

This theme discusses that social relationship networks within ethnic community are categorized into community resident networks and ethnic minority networks based on membership criteria. These networks serve a critical function in providing informational support for patients’ healthcare decision-making, though their operational paradigms differ significantly across relational fields.

In community resident networks, informational support predominantly manifests through spillover mechanisms, which enhance social actors’ action efficiency and success rates by disseminating knowledge beyond immediate interaction circles. Conversely, ethnic minority networks exhibit limited cross-network informational mobility. Within these endogenous relational fields, informational resources undergo integrated utilization during social actors’ decision-making processes, achieving support effects through collective reinforcement rather than external diffusion.

Participant 4, an out-of-town visitor, sought medical care at Community Health Center due to exacerbated cold symptoms during initial acclimatization to the local environment.

*“As soon as I got out of the car, I felt dizzy. I’d already been feeling off earlier, so I grabbed some meds, but guess they did not work – maybe I’ve been pushing myself too hard lately. When checking into the hotel, I asked the owner about nearby clinics. He said the Community Health Center was close and the medical care there was decent, so that’s where I went.*

*“After the treatment, I actually felt better. The doctor was super thorough – asked when my symptoms started, what meds I’d taken, if I had any allergies, all that stuff. Nurses were really attentive too. Overall it was a solid experience. Like, if I were just shopping I probably would not take the owner’s word for it, but when you are sick? Yeah, you kinda trust locals’ advice. Feels different when it’s your health on the line.”(participant 4).*

In the medical diagnostic and therapeutic process, an inherent asymmetry exists in the distribution and comprehension of medical information between healthcare providers and patients (37), which directly impacts the establishment of physician-patient trust and patients’ evaluation of therapeutic outcomes. As a non-local visitor, Participant 4 demonstrated limited awareness of medical resources in the ethnic community. To mitigate potential deficiencies in healthcare decision-making stemming from informational disparities, she sought assistance from local residents and subsequently selected Community Health Center as her treatment facility based on intelligence gathered from the community’s social network.

This process of acquiring informational support from the endogenous social relational network of local residents illustrates how collaborative social actions between Participant 4 and community members mitigated boundary constraints between in-group and out-group populations. The informational flow from the internal relational network to external individuals manifested through a spillover effect within the network field, thereby facilitating informational support and enabling social actors to access relevant societal resources. The positive therapeutic outcome and resolution of Participant 4’s medical condition following treatment at the selected institution substantiate the validity and reliability of information embedded within community relational networks.

Distinct from general community networks, ethnic minority relational networks demonstrate heightened interpersonal cohesion influenced by ethnic identity. Information circulating within these ethnically-bound networks exhibits distinct ethnic characteristics: Primarily, informational support within ethnic networks operates through systematic integration and utilization of intelligence within the relational field. Secondarily, ethnic minority patients frequently manifest a preference for seeking medical care from co-ethnic practitioners.

Participant 5, a local ethnic minority resident, derived his healthcare information through an intranet-based ethnic relations social network,

*“We, the villagers here (from the Buyi ethnic group), mostly go to our local clinic for medical care. The elders, young folks – pretty much everyone comes here. Taking medicine on our own can only do so much compared to getting help from real doctors. I’ve lived here my whole life, and I know some of the doctors there personally. Some are actually from our own village, which makes us trust them even more. The treatments work fine most of the time, and if things get too serious, they’ll refer us to a bigger hospital. Never heard of any medical mishaps happening here. Everybody in our community relies on this health center for regular illnesses. “(Participant 5).*

Participant 5’s selection of Community Health Center as their healthcare institution stems from two principal factors rooted in the local ethnic minority social context. Firstly, prolonged communal interactions have facilitated effective integration and utilization of medical institutional information within the ethnic minority relational networks, thereby generating robust informational support for minority patients. Secondly, the presence of co-ethnic medical practitioners enhances the perceived reliability of therapeutic services, significantly increasing the probability of healthcare facility selection.

Through ethnographic observation, it was documented that Participant 5 consistently employed their native minority language during clinical consultations. In rural ethnic minority communities, linguistic congruence serves dual functions: it enables clinicians to obtain precise clinical data during diagnostic processes, thereby improving treatment efficiency, while simultaneously fostering social proximity through shared ethnic identity. This unimpeded physician-patient communication constitutes a critical precondition for establishing constructive clinical relationships.

The longitudinal accumulation and systematization of medical information within ethnic relational networks provides substantive decision-making support for healthcare choices. Concurrently, ethnolinguistically constructed ethnic identity emerges as a pivotal factor facilitating positive clinical interactions. These dual mechanisms - informational support and identity-mediated relational dynamics - collectively shape patients’ healthcare-seeking behaviors.

In summary, two predominant social relational networks within ethnic community demonstrate differentiated impacts on medical decision-making processes. Distinct patterns emerge across relational network domains due to structural constraints affecting information mobility and variations in resource integration capabilities. Under the influence of ethnic relational dynamics, minority patients exhibit preferential selection of co-ethnic physicians, a phenomenon attributable to enhanced therapeutic efficacy perception and embedded trust mechanisms. This pattern further substantiates the guiding role of interpersonal trust in patients’ selection of informal healthcare institutional arrangements.

### 3.3 Theme3: healthcare-seeking behavior under cultural norms and institutional norms

This theme mainly discusses how cultural norms and institutional norms affect the healthcare-seeking behaviors process of patients. Social norms represent behavioral standards widely accepted and endorsed by community members, capable of guiding or constraining individual behaviors in the absence of legal enforcement (38). These norms function dually as prescriptive frameworks for social actors while simultaneously providing behavioral safeguards. In the healthcare-seeking practices of ethnic minority villagers in ethnic community, social norms manifest as an interplay between institutional regulations and cultural prescriptions, with distinct normative systems offering therapeutic assurances during patients’ selection processes across different medical paradigms.

Participant 6, an extra-local patient seeking ethnomedical healthcare, presented with severe hand burns. Following referrals from local Buyi ethnic residents in the ethnic community, he sought therapeutic services from a traditional ethnic healer——participant7.

*“Back then, right after I got burned at the factory, I rushed straight to the local hospital for emergency care. They did basic first aid and bandaged me up, but the burns were pretty gnarly. The docs said I might even need skin grafts, and that the medical bills were gonna pile up big time. Luckily, this auntie from our family—you know, the one who married into that Buyi ethnic village—heard about this traditional healer guy. Turns out he was a Bumuo (a traditional healer-priest from the Buyi people) who ran this treatment spot, and people swore his methods worked. So we hauled ass over there. Those Bumuo folks are like super respected in their community, right? “(participant 6).*

In traditional ethnic communities within China, modern healthcare systems may confront dual challenges: underdeveloped medical infrastructure and delayed dissemination of contemporary medical knowledge. This constellation of challenges shapes social capital within healthcare processes—manifested through differential trust attitudes and information-sharing networks. Concurrently, healthcare accessibility and treatment-related expenses (7) significantly influence residents’ healthcare-seeking decisions. The health-seeking trajectory of Participant 6 exemplifies the functional substitutability between traditional ethnic medical systems and modern biomedical systems within specific socioeconomic contexts. Furthermore, participant6’s therapeutic course reveals the profound impact of cultural norms on the medicinal decision-making processes of Buyi traditional healers.

*“The treatment has been working really well. When I first started coming here, my entire hand would hurt like crazy every day, so I was pretty worried. But the Bumo (the ethic healer) kept reassuring me. After the ethic healer worked on my hand, I could feel this cool sensation spreading through it. Whenever they changed the dressings, they’d check in about my recovery progress and even gave me a rough idea of when I’d recover. Honestly, it gave me peace of mind knowing their treatments followed a proper system - like they really knew what they were doing every step of the way.”(participant 6).*

*“Treating patients is something I’ve got my own approach to. A lot of the folks I see are from my own Buyi ethnic group, and sticking to proper procedures during treatment really makes a difference in their experience. When I’m working with someone, I always chat with them about how they are feeling—both about their illness and the treatment plan. See, our traditional medical texts do not just list herbs; they also include real-life cases. All this passed-down medical knowledge stresses treating the whole person—mood and mindset included—to hit that sweet spot where patients feel good inside and out. Stuff they are stressed about, like money worries or how long healing’ll take? Gotta walk through all that detail by detail with them.” (Participant 7).*

The Buyi traditional medicine system in the ethnic community demonstrates notable recognition, primarily manifesting in therapeutic modalities including herbal poultices, medicinal wines, and acupuncture, with particular specialization in burn management and traumatic injury treatment. Practitioners identify themselves as Bumo (ritual specialists) within the Buyi ethnic tradition, deriving their therapeutic expertise from the Mojing Scriptures - sacred texts documenting Buyi shamanic healing practices and phytotherapeutic applications using traditional Buyi script.

The cultural ascendancy of this medical paradigm stems not solely from therapeutic efficacy but profoundly from its embeddedness in Buyi medical cosmology. Notably, sociocultural factors exert significant influence on healthcare-seeking behaviors, particularly manifest in patients’ preference for Buyi ethnomedical interventions. Central to this medical culture persists the principle of “dual resolution paradigm: addressing psycho-spiritual crises through biomedical and existential therapeutics” (shen yao liang jie) and “integration of shamanism and medicine” (wu yi he yi). Clinical protocols systematically incorporate patients’ spiritual beliefs and cultural frameworks, thereby enhancing therapeutic outcomes through holistic syncretism.

This medical philosophy imposes expanded cultural obligations upon Bumo practitioners, necessitating dual attention to somatic pathology and psychospiritual states. Empirical observations confirm that burn and trauma patients in the community predominantly utilize this ethnomedical service.

In contrast to practitioners within informal healthcare systems, whose professional conduct is predominantly shaped by cultural norms, physicians operating under formal healthcare institutions are obligated to not only adhere to sociocultural conventions but also rigorously implement institutional protocols while maintaining systemic credibility.

*“We nurses must do our utmost for patients. Not only us nurses, but all the staff in the hospital hope that patients can be cured. According to the regulations set by the hospital director, we should ensure that patients have living conditions equal to ours. If it’s mealtime, patients still receiving treatment at the hospital should also eat with us. Moreover, none of us here are wealthy, so we need to consider the actual situation of the patients when it comes to medication specifications and prices. Many older adult people do not understand the process of medical insurance reimbursement, and they need our patient guidance. We will do our best to see every patient leave the hospital healthy and well.”(participant 8).*

Analysis of Participant 8’s discourse reveals that sociocultural norms impose stringent regulatory expectations on physicians within formal healthcare systems, particularly emphasizing the imperative to maintain optimal physician-patient rapport. In contrast, institutional norms exhibit broader universality in scope. Substantively, these regulations mandate that physicians extend care beyond clinical treatment protocols to encompass holistic consideration of patients’ daily lives, as exemplified by stipulations such as “cohabitation and shared daily activities” with patients. Formally, institutional norms are codified not only in written policies but also manifested through oral traditions and customary behavioral guidelines. This multifaceted norm framework ensures patient confidence in seeking treatment at Community Health Center, thereby solidifying its status as the township’s most frequented medical facility. Consequently, systematic institutional norm constitutes a critical determinant of physician conduct in formal healthcare systems and a foundational pillar of patient care quality. Compliant physician practices engender optimized therapeutic processes and outcomes, while institutional norms—as elements of the regulatory environment—directly shape patient trust in formal healthcare systems, ultimately influencing care-seeking decisions.

In summary, distinct social norms within the community’s ethnic minority rural context yield divergent effects across healthcare systems. In informal healthcare systems, practitioners of ethnomedicine (e.g., Buyi traditional medicine) operate under the constraints of Buyi cultural norms. Conversely, formal healthcare institutions require physicians to adhere to dual normative frameworks: sociocultural expectations and institutional regulations, with the latter demonstrating greater universality. Both norm paradigms jointly ensure procedural and efficacy safeguards for patient care, which critically inform patient trust attitudes during healthcare facility selection. Such trust subsequently functions as a behavioral influential in care-seeking pathways.

## 4 Discussion

The community context exerts a profound influence on the lived experiences of residents within ethnic minority rural settlements. Within the sociostructural milieu of these communities, the impact of social capital on healthcare-seeking behaviors manifests through three dimensions: social trust, social networks, and social norms, all of which shape patient healthcare seeking decision-making processes. Under conditions of societal transition, the interplay between formal and informal healthcare systems, institutional frameworks and cultural practices, as well as traditional and modern medicine, becomes acutely visible in the healthcare choices of rural ethnic minority populations and the mediating role of social capital.

First, formal healthcare systems governed by administrative-institutional logic maintain relative dominance in residents’ healthcare decisions. This primacy stems from professionalized therapeutic protocols and externally imposed authoritative forces. In contrast, informal healthcare systems rooted in sociocultural logic demonstrate enduring vitality, serving as critical complements to—and occasionally substitutes for—formal systems, particularly in managing culturally specific health conditions.

Second, patients’ selection of healthcare systems reflects dynamic institution-culture interactions. While modern institutional frameworks appear to wield greater pragmatic utility in contemporary society, particularly in guiding clinical judgment and decision-making processes, sociocultural forces retain significant agency. This is evidenced not only through patient preferences for informal systems but also through practitioners’ adherence to culturally embedded therapeutic norms. Crucially, such cultural resilience persists rather than diminishes amid institutional modernization, maintaining functional relevance during societal transitions (39).

Finally, traditional medical practices continue to carve developmental niches despite the ascendancy of modern biomedicine. As exemplified by ethnomedical systems referenced earlier, which have evolved through therapeutic praxis that is transmitted across generations, innovative paradigms like “The Dual Resolution Paradigm: Addressing Psycho-Spiritual Crises Through Biomedical and Existential Therapeutics” demonstrate the viability of traditional healing. Such approaches foreground patient-centered illness narratives and somatic-semiotic expressions of distress, offering critical insights into sustaining traditional medicine’s vitality within modernization-driven societies. Their enduring relevance is anchored in empirically demonstrated efficacy in resolving patients’ psychospiritual crises—a therapeutic dimension frequently under-addressed by conventional biomedicine (40). The societal role of the Buyi ethnic healer, designated as a “ritual healer,” not only establishes specific cultural norms during therapeutic processes but also demonstrates the Buyi ethnomedical cultural system’s validation of pathology and therapeutic efficacy through patient recovery. This is achieved through the alignment between the patient’s illness narrative framework and the cultural norms of traditional healing practitioners. Such congruence mitigates disease-related societal discomfort while fostering patient adherence to traditional medical practices (41).

Returning to this study’s exploration of how social capital influences residents’ healthcare-seeking behaviors, the uniqueness of social capital as a context-dependent concept in ethnic minority rural communities manifests particularly through interethnic networks and the consequent trust minority patients place in co-ethnic physicians (42). Social capital exerts multifaceted roles including dominance, support, and safeguarding in shaping patients’ healthcare selection behaviors, reflecting the intricate relationship between social capital and health – wherein social capital influences health-related behaviors through diversified pathways (24).

The social capital-health nexus discussed herein carries significant implications for refining China’s health policies. Adjustments to medical policies such as the hierarchical medical system require coordinated implementation with grassroots hospital initiatives. The autonomous actions of primary healthcare institutions demonstrate greater contextual relevance than policy directives in influencing patient choices, as medical facilities with higher social capital typically attract greater patient volumes. Concurrently, informal medical institutions could serve as complementary forces to formal healthcare systems, providing patients with diversified yet effective alternatives. This necessitates not merely policy realignment but also reserved developmental space for informal medical mechanisms to harmonize rural healthcare development, preserving elements conducive to rural health advancement during modernization. Ultimately, health policy reformulation must anchor itself in patients’ lived realities and social ecosystems to authentically unleash the transformative potential of health governance.

## Data Availability

The original contributions presented in the study are included in the article/supplementary material, further inquiries can be directed to the corresponding author.

## References

[1] National Health Commission of the People's Republic of China. (2023). China health and health statistics yearbook (2024). Available online at: http://www.nhc.gov.cn/mohwsbwstjxxzx/tjtjnj/202501/b8d57baa95834269b5b3562bfec801a7.shtml (Accessed April 1, 2025).

[2] Athumani KF Mboineki JF. Healthcare facility factors associated with health-seeking behavior among secondary school students in the Dodoma region: an analytical cross-sectional study. Front Public Health. (2025) 13:1457318. doi: 10.3389/fpubh.2025.1457318, PMID: 40213432 PMC11983427

[3] Siyang S. Factors influencing the medical behavior of rural elderly-An empirical study based on data from Gucheng and Nanyang. Population and Development. (2016) 5:69–74+60. doi: 10.3969/j.issn.1674-1668.2016.05.008

[4] Widayanti AW Green JA Heydon S Norris P. Health-seeking behavior of people in Indonesia: a narrative review. J Epidemiol Glob Health. (2020) 1:6–15. doi: 10.2991/jegh.k.200102.001 PMC7310809 32175705

[5] Musoke D Boynton P Butler C Musoke MB. Health seeking behaviour and challenges in utilising health facilities in Wakiso district, Uganda. Afr Health Sci. (2014) 14:1046–1055. doi: 10.4314/ahs.v14i4.36, PMID: 25834516 PMC4370086

[6] Benfeng Du D Feng M. Factors influencing the choice of medical treatment destinations for young migrant population and measurement analysis -Based on surveys in Beijing, Shanghai, and Shenzhen. Popul Res. (2012). 6:71–86.

[7] Andersen R Aday LA. Access to medical care in the US: realized and potential. Med Care. (1978) 16:533–46. doi: 10.1097/00005650-197807000-00001, PMID: 672266

[8] Wu F Wang N Qu Y. Where does residents' choice of primary medical treatment come from?—a logical analysis based on the perspective of service accessibility and residents' cognition. Front Public Health. (2022) 10:949622. doi: 10.3389/fpubh.2022.949622, PMID: 36324459 PMC9618942

[9] Qiu C Zhang Y Wang X Gu D. Trust-based research: influencing factors of patients’ medical choice behavior in the online medical community. Healthcare. (2022) 5:98. doi: 10.3390/healthcare10050938, PMID: 35628075 PMC9140699

[10] Shaikh BT Hatcher J. Health seeking behaviour and health service utilization in Pakistan: challenging the policy makers. J Public Health. (2005). 1:49–54. doi: 10.1093/pubmed/fdh207 15590705

[11] Brown PH Theoharides C. Health-seeking behavior and hospital choice in China's new cooperative medical system. Health Econ. (2009) 2:47–64. doi: 10.1002/hec.1508 19551751

[12] Westin M Åhs A Bränd Persson K Westerling R. A large proportion of Swedish citizens refrain from seeking medical care—lack of confidence in the medical services a plausible explanation? Health Policy. (2004) 68:333–44. doi: 10.1016/j.healthpol.2003.10.008, PMID: 15113644

[13] Macq J Solis A Ibarra M Martiny P Dujardin B. The cost of medical care and people's health-seeking behaviour before being suspected of tuberculosis in three local health systems, Nicaragua. Int J Tuberc Lung Dis. (2000) 11:1330–1336. doi: 10.1016/j.respol.2010.05.003 15581201

[14] Thorson A Hoa NP Long NH. Health-seeking behaviour of individuals with a cough of more than 3 weeks. Lancet. (2000) 9244:1823–1824. doi: 10.1016/S0140-6736(00)03241-4, PMID: 11117921

[15] Beiersmann C Sanou A Wladarsch E de Allegri M Kouyaté B Müller O. Malaria in rural Burkina Faso: local illness concepts, patterns of traditional treatment and influence on health-seeking behaviour. Malar J. (2007) 6:1–9. doi: 10.1186/1475-2875-6-106, PMID: 17686147 PMC1971712

[16] Feng X Hu Y Pfaff H Liu S Wang H Qi Z. The determinants of help-seeking behaviors among cancer patients in online health communities: evidence from China. Int J Med Inform. (2025) 195:105767. doi: 10.1016/j.ijmedinf.2024.105767, PMID: 39721114

[17] Bourdieu P. The forms of capital In: Richardson J, editor. Handbook of theory and research for the sociology of education. New York: Greenwood. (1986).

[18] Coleman JS. Foundations of social theory. Cambridge, MA: Harvard University Press. (1980).

[19] Putnam R Leonardi R Nanetti R. Making democracy work: Civic tradition in modern Italy. Princeton, NJ: Princeton University Press. (1993).

[20] Lin N. Social capital: A theory of social structure and action. Cambridge, England: Cambridge University Press. (2001).

[21] Bian Y. Bringing strong ties back in: indirect ties, network bridges, and job searches in China. Am Sociol Rev. (1997) 62:366–85. doi: 10.2307/2657311

[22] Yang M. Gifts, favors and banquets: The art of social relationships in China. Ithaca, NY: Cornell University Press (1994).

[23] Zhou G Fan G Shen G. Income disparities, social capital and health —— an empirical analysis based on the China family panel studies (CFPS). Managing the World. (2014) 2014:07. doi: 10.19744/j.cnki.11-1235/f.2014.07.004

[24] Kawachi I Subramanian SV Kim D. Social capital and health: A decade of progress and beyond. New York, NY: Springer. (2008).

[25] Kawachi I Kennedy BP. Income inequality and health: pathway and mechanism. Health Serv Res. (1999):34. PMC1088996 10199670

[26] Poortinga W. Social relations or social capital? Individual and community health effects of bonding social capital. Soc Sci Med. (2006) 63:255–70. doi: 10.1016/j.socscimed.2005.11.039, PMID: 16427171

[27] Kawachi I. Social capital and community effects on population and individual health. Ann N Y Acad Sci. (1999) 896:120–130. doi: 10.1111/j.1749-6632.1999.tb08110.x, PMID: 10681893

[28] d'Hombres B Rocco L Suhrcke M McKee M. Does social capital determine health? Evidence from eight transition countries. Health Econ. (2010) 1:56–74. doi: 10.1002/hec.1445, PMID: 19301350

[29] Kawachi I Kennedy BP Glass R. Social capital and self-rated health: a contextual analysis. Am J Public Health. (1999) 89:1187–1193. doi: 10.2105/ajph.89.8.1187, PMID: 10432904 PMC1508687

[30] Saliby E. Descriptive sampling: a better approach to Monte Carlo simulation. J Oper Res Soc. (1990) 41:1133–1142. doi: 10.1057/jors.1990.180

[31] Koenigsmann M Koehler K Regner A Franke A Frommer J. Facing mortality: a qualitative in-depth interview study on illness perception, lay theories and coping strategies of adult patients with acute leukemia 1 week after diagnosis. Leuk Res. (2006) 30:1127–34. doi: 10.1016/j.leukres.2005.12.016, PMID: 16458356

[32] Dunzin NK Lincoln YS In: Feng XT, editor. Qualitative research: Methodological foundations: Volume 1. Chongqing: Chongqing University Press (2007)

[33] Lijie F. The formation process of institutional trust —— taking the new rural cooperative medical system as an example. Sociol Stud. (2009) 2:130–148. doi: 10.19934/j.cnki.shxyj.2009.02.007

[34] Anyan F Andoh-Arthur J Adjei SB Akotia CS. Mental health problems, interpersonal trust, and socio-cultural correlates of corruption perception in Ghana. Front Public Health. (2024) 12:1269579. doi: 10.3389/fpubh.2024.1269579, PMID: 38481830 PMC10933067

[35] Kleinman A In: Xiaoli F, editor. The story of illness. Shanghai: Shanghai Translation Publishing House (2018)

[36] Siqi J Chunguang W. Research on the structural basis and logic of the village multi-level medical system. Sociol Stud. (2022). 1:46-67+227.

[37] Ramírez AS Estrada E Ruiz A. Mapping the health information landscape in a rural, culturally diverse region: implications for interventions to reduce information inequality. J Prim Prev. (2017) 38:345–62. doi: 10.1007/s10935-017-0466-7, PMID: 28224349 PMC5478445

[38] Ostrom E. Collective action and the evolution of social norms. J Econ Perspect. (2000) 3:137–158. doi: 10.1080/19390459.2014.935173

[39] Cruz ML Christie S Allen E Meza E Nápoles AM Mehta KM. Traditional healers as health care providers for the Latine community in the United States, a systematic review. Health equity. (2022) 6:412–26. doi: 10.1089/heq.2021.0099, PMID: 35801152 PMC9257545

[40] Nortje G Oladeji B Gureje O Seedat S. Effectiveness of traditional healers in treating mental disorders: a systematic review. Lancet Psychiatry. (2016) 3:154–70. doi: 10.1016/s2215-0366(15)00515-5, PMID: 26851329

[41] Finkler K. Women in pain: Gender and morbidity in Mexico. Philadelphia, PA: University of Pennsylvania Press. (1994).

[42] Alecu AI. Which doctors do we trust? A vignette experiment of how gender and ethnicity influence trust. Ethnicities. (2020) 20:939–58. doi: 10.1177/1468796819877737

